# Diagnosing Hyper IgE Syndrome in a Resource‐Limited Setting: A Case Report Highlighting the Utility of Clinical Scoring and Multidisciplinary Management

**DOI:** 10.1155/crii/5929061

**Published:** 2026-07-03

**Authors:** Roshna Devi, Huda Raja, Prithvi Raj, Muhammad Hassan Ahsan, Abdullah Nadeem, Nahid Raufi

**Affiliations:** ^1^ Department of Pediatrics, Dow University of Health Sciences, Karachi, Pakistan, duhs.edu.pk; ^2^ Department of Medicine, Dow University of Health Sciences, Karachi, Pakistan, duhs.edu.pk; ^3^ Department of Medicine, Kabul Medical University, Kabul, Afghanistan

**Keywords:** elevated IgE, genetic counseling, hyperimmunoglobulin E syndrome, Job’s syndrome, MRSA, pediatric immunodeficiency, primary immunodeficiency, pustular lesions, recurrent infections, suppurative lymphadenitis

## Abstract

**Background:**

Hyperimmunoglobulin E syndrome (HIES), also known as Job’s syndrome, is an uncommon primary immunodeficiency disorder characterized by markedly elevated serum IgE levels, recurrent infections, both bacterial and fungal, and distinct skeletal and connective tissue anomalies. This report discusses the case of a 3‐year‐old female patient who presented with a history of recurrent chest infections, pustule formation, and local abscesses, and investigation revealed high levels of IgE, and she was eventually diagnosed with HIES.

**Case Presentation:**

A 3‐year‐old girl from Karachi presented with a 1‐year history of recurrent pustular skin eruptions initially involving the scalp and face, later spreading across the body. The lesions appeared in clusters, drained pus, resolved with pigmentation changes, and recurred every few weeks. She also had recurrent fever, episodes of diarrhea, frequent upper respiratory tract infections (URTIs), and poor weight gain. Her past treatment included multiple oral antibiotics and incision and drainage of an axillary abscess. On examination, she appeared pale, undernourished, and had widespread pustular lesions and localized abscesses, particularly over the parotid and neck regions, along with palpable cervical lymphadenopathy. Laboratory findings showed microcytic anemia, eosinophilia, leukocytosis, elevated ESR (erythrocyte sedimentation rate), and significantly raised serum IgE (7162 IU/mL), with normal IgG, IgM, and IgA levels. Methicillin‐resistant *Staphylococcus aureus* (MRSA) was isolated from skin cultures, *E. coli* from blood culture, and *Giardia lamblia* from stool. A lymph node biopsy confirmed suppurative lymphadenitis. Based on clinical features and immunological profile, a diagnosis of Hyper IgE Syndrome was made, although genetic confirmation could not be obtained due to limited resources.

**Conclusion:**

Early recognition is essential to prevent complications, guide appropriate management, and offer genetic counseling. This case emphasizes the importance of maintaining a high index of suspicion in patients with recurrent infections.

## 1. Introduction

Hyperimmunoglobulin E syndrome (HIES), also known as Job’s syndrome, is an uncommon primary immunodeficiency disorder characterized by markedly elevated serum IgE levels, recurrent infections, both bacterial and fungal, and distinct skeletal and connective tissue anomalies [1]. It may follow either an autosomal dominant or recessive inheritance pattern, with the dominant form most frequently associated with mutations in the STAT3 gene [2]. The estimated prevalence of HIES is ~1 in 100,000 individuals, with an incidence of less than 1 per million annually, and no clear gender predilection has been observed [2, 3]. In nearly 78% of cases, HIES manifests early in life with a rash that resembles atopic dermatitis. This cutaneous eruption typically begins as erythematous papules that evolve into pustules, which subsequently exude and form crusts. Unlike classic atopic dermatitis, scaling and lichenification are usually mild or absent. The rash is often pruritic due to histamine release from mast cells activated by IgE and tends to appear on the scalp, face, neck, axillae, and diaper region [1].

Certain features help differentiate HIES‐related dermatitis from atopic dermatitis, including its early onset, prolonged and severe course, atypical distribution (particularly in the axillae, groin, and perineum), frequent superimposed *Staphylococcus aureus* infections, formation of cold abscesses, poor response to conventional therapies, and notable improvement with antistaphylococcal antibiotics [4]. Additional systemic findings often seen in HIES include eczema‐like dermatitis, recurrent abscess formation, pulmonary infections complicated by pneumatoceles, and chronic mucocutaneous candidiasis [5]. Other characteristic features may include coarse facial features, delayed exfoliation of primary teeth, spinal deformities such as scoliosis, and joint hyperextensibility [6]. The broad clinical spectrum of HIES frequently leads to delayed or missed diagnoses, underscoring the need for increased clinical awareness and timely genetic evaluation.

This report discusses the case of a 3‐year‐old female patient who presented with a history of recurrent chest infections, pustule formation, and local abscesses, and investigation revealed high levels of IgE, and she was eventually diagnosed with HIES. The case highlights diagnostic challenges and the critical role of clinical suspicion supported by immunological and genetic evaluations.

## 2. Case Report

A 3‐year‐old girl, who was born electively via C‐section, fully vaccinated with a weight of 10 kg, a resident of Karachi, presented to our emergency with complaints of pustular lesions for 1 year. She started developing pustular lesions (rashes) involving her head, forehead, cheeks, and neck and then spread to the whole body over a period of 1 month. It erupted in clusters and dried up in 2–3 weeks, followed by the next set of eruptions after 2–3 weeks. Her rashes were pruritic, pus‐draining and were associated with altered pigmentation (both hypo and hyper) after resolution. It was also noted that recurrent formation of localized pus‐containing swellings occurred over the skin of the head, face, and neck region, unrelieved by any ointments or lotions. It was also associated with fever; intermittent, high‐grade, documented (102°F), 3–4 spikes per day, relieved temporarily by OTC (over‐the‐counter) antipyretics but not associated with rigors, chills, or seizures, and no specific time distribution of spikes was noted. Systematic examination revealed a positive history for recurrent diarrhea, chest infections (upper respiratory tract infection [URTI]) and rhinitis. It was noted that her episodes of recurrent diarrhea were of porridge consistency, 5–6 times per day, an episode lasting for 4–5 days with recurrence every 1–2 months. Over the preceding year, she had visited general practitioners on eight occasions because of a dry cough, purulent nasal discharge, and fever. During those visits, she received multiple courses of oral antibiotics and underwent incision and drainage of an axillary lymph node abscess. Her caloric intake was estimated to be only about 50% of the age‐appropriate requirement, largely due to recurrent infections, chronic diarrhea, and poor appetite (socioeconomic factors were not formally assessed). There was no family history of similar complaints.

On examination, a pale‐looking female child with a thin, lean build without any dysmorphic features was conscious but irritated and had multiple pustular lesions involving the head, neck, face, and whole body. She was vitally stable, anemic‐looking, afebrile with blood pressures in the 50th percentile, with a weight of 10 kg (5^th^ cent). Her anthropometrics were in the SD score of less than −2 [height of 92 cm (75^th^ cent), OFC of 48 cm, mid‐arm circumference of 11.8 cm]. Another significant finding was palpable bilateral submandibular lymph nodes measuring ~0.5–1 cm. Her skin showed multiple pustular lesions involving her entire head, forehead, and cheeks with localized abscesses over the right parotid region, 2 cm × 3 cm, and on the right side of the neck, 2 cm × 2 cm, with suppurative discharge and crusting noted around the margins. Pustular lesions over the thorax, upper limbs, lower limbs, upper trunk, and back were about 0.5 cm in size. Multiple scarred areas of hypo and hyperpigmentation all over the body (healed lesions). Otherwise, her examination of the chest, circulatory system, and abdomen was normal.

Her investigations revealed microcytic, hypochromic anemia with Hb of 7.6 mg/dL and eosinophilia (109/L) and leucocytosis with predominance of neutrophiles (66.9%). Erythrocyte sedimentation rate (ESR) was raised to 25 mm/h and AEC (absolute eosinophil count) of 2772 cells/µL. A repeat AEC on hospital day 8 was 2450 cells/µL, indicating persistent eosinophilia throughout the admission. Anti‐TTG IgA was done 2 months back, which came back negative (Table 1). A blood CS was sent during a recent fever spike, which was *E*.*coli* positive, sensitive to piperacillin and meropenem. Pus culture revealed MRSA (methicillin‐resistant *Staphylococcus aureus*) sensitive to multiple common antibiotics, including clindamycin, erythromycin, etc. Stool examination revealed cysts of *Giardia Lamblia*. Excisional biopsy of the submandibular nodes was done, which showed suppurative lymphadenitis with abscess formation (Table 2). Immunological workup showed IgE levels of 7162, while IgG, IgM, and IgA were normal, and HIV screening was negative. An immunologist was taken on board who suspected a high possibility of HIES, and genetic analysis was advised, but unfortunately, it could not be done due to a lack of resources. As per the National Institutes of Health (NIH) score (7), a 10 score for IgE level (>2000), 4 for abscess (3–4), 0 for pneumonia, parenchymal lung anomalies, retained primary teeth, characteristic face, midline anomaly, newborn rash, candidiasis, scoliosis, and fractures, 1 for eczema, 6 for eosinophilic count, 2 for URTI, and 3 points for young‐age correction (2–5 years), with a total score of 26 points. The final diagnosis of HIES was made.

**Table 1:** Hematological and biochemical findings.

Parameter	Result	Normal range
Hemoglobin	7.6 mg/dL	11.5–15.5 mg/dL
Total leukocyte count	Elevated	4000–11,000/mm^3^
Neutrophils	66.9%	40%–60%
Eosinophils	109/L	<500/mm^3^
ESR	25 mm/h	<20 mm/h
Absolute eosinophil count (AEC)	2772/mm^3^	<500/mm^3^
IgE	7162 IU/mL	<150–300 IU/mL
IgG, IgM, and IgA	Normal	—
Anti‐TTG IgA	Negative	Negative
HIV	Negative	—

**Table 2:** Microbiological and pathological findings.

Test	Result
Blood culture	*E. coli* (sensitive to piperacillin, meropenem)
Pus culture	MRSA (sensitive to clindamycin, erythromycin)
Stool examination	*Giardia lamblia* cysts
Lymph node biopsy	Suppurative lymphadenitis

**Figure 1:**
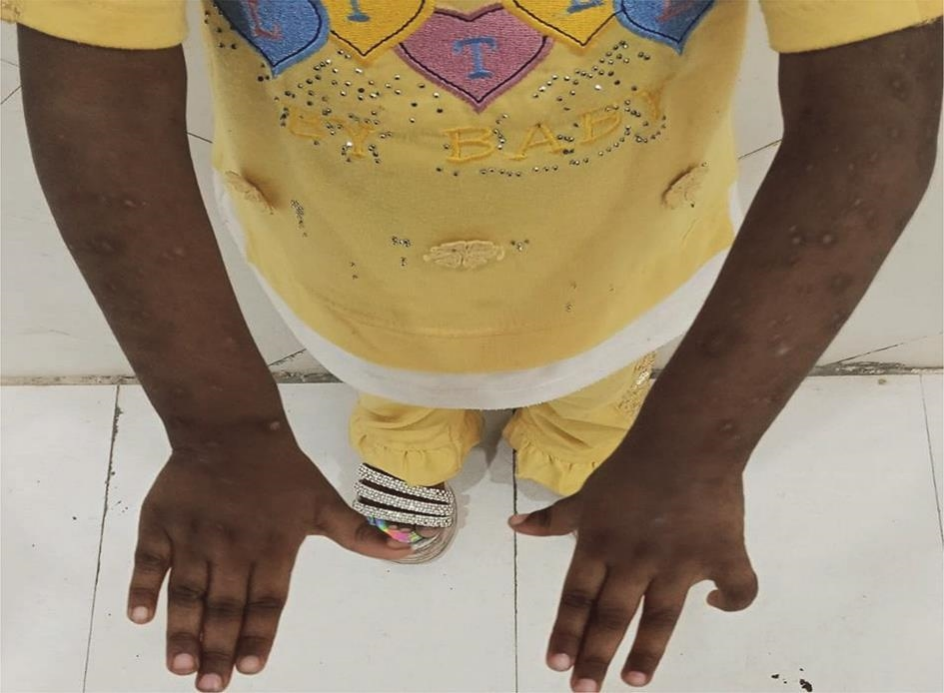
Posttreatment improvement in pustular eruptions in bilateral upper limbs.

**Figure 2:**
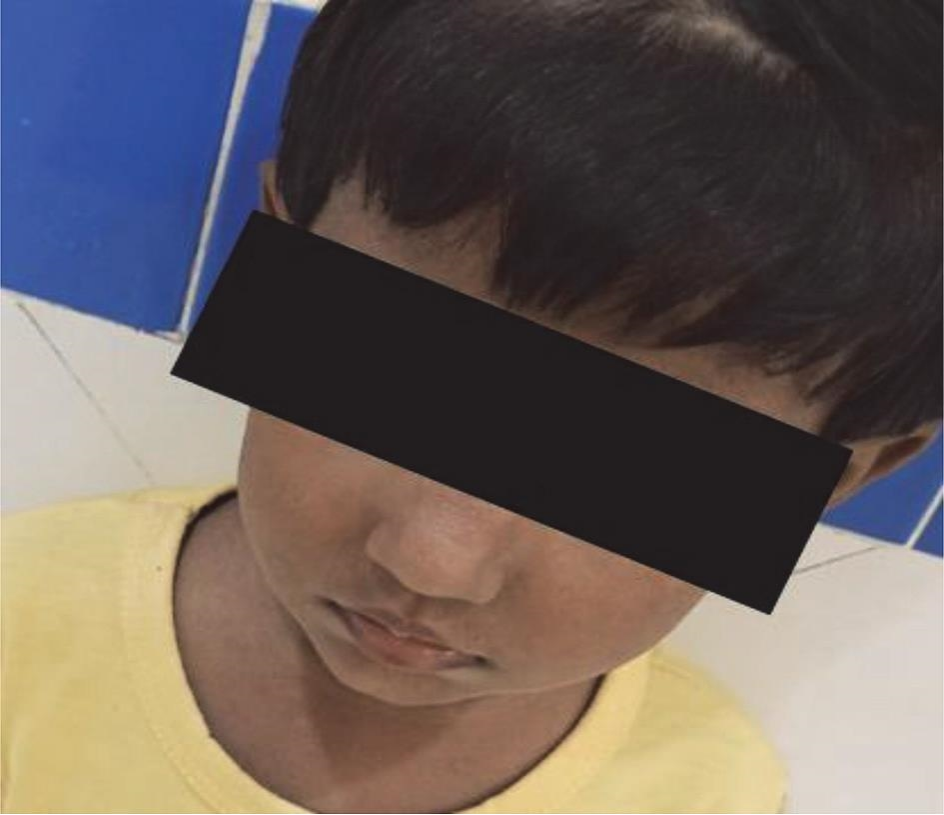
Posttreatment facial improvements.

Her treatment included piperacillin and tazobactam (100 mg/kg/dose) and oral metronidazole (15 mg/kg/day) given every 8 h, with topical application of fusidic cream thrice daily and vitamin D, Zinc, and B‐complex supplementation. Fever was managed with oral paracetamol suspension and sponging. Cetirizine was given orally to manage allergic symptoms. Her skin was managed with dermatological advice, which included skin hydration, hygiene maintenance with chlorinated water baths (½ bleach in 1 tub of water for 15 min) for 2–3 times a week, and incision and drainage of abscesses. Her hospital stay was for 11 days, and on the 8th day, a repeat blood culture was sent, which came out to be negative. Her skin condition had improved, and she was then discharged on multivitamin supplementation and was counselled for good hygiene along with sun protection. She was followed up in the pediatrics OPD after 1 week of discharge. She was free from eruptions and abscesses, and her itching was drastically improved, while the altered pigmentation persisted. Her parents were advised to follow up with the patient on an outpatient basis once per month for 6 months if no acute issue occured. Figure 1 shows posttreatment improvement in pustular eruptions in bilateral upper limbs. Figure 2 shows posttreatment facial improvements.

## 3. Discussion

HIES is a rare and often under‐recognized primary immunodeficiency with diverse clinical manifestations that can mimic more common conditions. Diagnosis can be problematic as there is no clear criterion, and symptoms are quite diverse, similar to our case. However, most clinicians diagnose HIES with its clinical features, and the scoring system is used to evaluate for STAT3 probability [7]. To aid in the clinical diagnosis of HIES in the absence of genetic confirmation, the NIH developed a scoring system that quantifies various clinical and laboratory features commonly associated with the syndrome. This tool is particularly useful in resource‐limited settings. The scoring system evaluates both major and minor features, including elevated serum IgE, eczema, recurrent infections (especially skin and lung abscesses), characteristic facial features, retained primary teeth, scoliosis, fractures, and eosinophilia. Each parameter is assigned a weighted score based on its diagnostic relevance, with a total score above 40 generally considered highly suggestive of STAT3‐mutated autosomal dominant HIES. However, lower scores in younger patients may still support a provisional diagnosis. In our patient, the total score was 26, which, though below the threshold for classical STAT3‐HIES, was significant in the context of her young age and clinical presentation. A score of 10 was assigned for IgE level (>2000), 4 for abscesses (3–4), 0 for pneumonia, parenchymal lung anomalies, retained primary teeth, characteristic face, midline anomaly, newborn rash, candidiasis, scoliosis, and fractures, 1 for eczema, 6 for eosinophilic count, 2 for URTI, and 3 points for young‐age correction (2–5 years) with a total score of 26 points (Table 3).

**Table 3:** NIH HIES scoring system and our patient’s score.

Feature	Possible score	Our patient’s finding	Score given
Serum IgE level >2000 IU/mL	10	7162 IU/mL	10
Recurrent skin abscesses	4	Present (3–4 episodes)	4
Recurrent pneumonia	4	Absent	0
Pathological lung findings (e.g., pneumatoceles)	4	Not observed	0
Retained primary teeth	2	Not applicable (age)	0
Characteristic facial appearance	3	Absent	0
Eczema (atopic dermatitis‐like rash)	4	Present	1
Eosinophilia (>700 cells/μL)	6	2772 cells/μL	6
Mucocutaneous candidiasis	2	Absent	0
Fractures with minimal trauma	2	Absent	0
Scoliosis	2	Not observed	0
Newborn rash	2	Absent	0
Midline abnormalities (e.g., palate)	2	Absent	0
Recurrent upper respiratory infections	2	Present	2
Age adjustment (2–5 years)	3	3‐years‐old	3
Total score	Max: 60+	—	26

A score of ≥40 is typically suggestive of STAT3‐associated HIES in older children and adults. However, in children under 5, lower scores may still be meaningful due to age‐adjusted expression of features. Our patient’s score of 26, combined with elevated IgE, eosinophilia, and recurrent infections, supports a probable clinical diagnosis of HIES, especially in the absence of other immunodeficiency syndromes.

We could not identify the mutations due to the unavailability of genetic testing for HIES in Pakistan and the financial disadvantage, which affected many other cases and has delayed diagnosis and treatment [8, 9]. Similar to the prior reported cases, we found a markedly raised level of IgE while the serum levels of immunoglobulins G, A, and M remained normal. However, in our case, we did not find eosinophilia up to 700 cells, like most cases; our patient had eosinophilia of 109/L [10].

The pathogenesis of autosomal dominant HIES is primarily driven by loss‐of‐function mutations in the STAT3 gene, which disrupt the signaling pathways of multiple cytokines, including interleukin‐6 (IL‐6) and IL‐23 [2, 5]. This impairment leads to defective differentiation of naïve CD4^+^ T cells into Th17 cells, resulting in markedly reduced production of IL‐17 and IL‐22 [2]. IL‐17 plays a critical role in recruiting neutrophils to sites of infection and inflammation; its deficiency explains the blunted inflammatory response observed in HIES patients, who often develop collections of pus that lack the classical warmth, erythema, and tenderness despite a heavy bacterial burden [5, 6]. In the present case, the recurrent deep‐seated abscesses with minimal surrounding inflammation are consistent with this immune defect. Although STAT3 mutational analysis could not be performed due to resource constraints, the clinical and immunological profile aligns closely with the established NIH‐HIES scoring system, which yielded a total score of 26 points, highly suggestive of STAT3‐deficient HIES [7]. This diagnostic approach is not uncommon in low‐resource settings; a very recent case report from Pakistan by Dave et al. [10] similarly relied on clinical criteria and markedly elevated IgE levels to diagnose probable HIES in the absence of genetic confirmation. Together, these cases underscore the ongoing challenge of definitive molecular diagnosis in regions with limited access to advanced genetic testing, while also highlighting the continued utility of composite clinical‐laboratory scoring systems for timely identification and management. DOCK8 deficiency (autosomal recessive HIES) typically presents with severe viral skin infections, low IgM, and marked eosinophilia, which our patient did not exhibit. Wiskott‐Aldrich syndrome is characterized by microthrombocytopenia, eczema, and recurrent infections; our patient’s normal platelet count and absence of bleeding tendency argue against it. Severe atopic dermatitis can elevate IgE, but lacks cold abscesses, pneumatoceles, and the characteristic infection pattern. The patient’s failure to respond to standard eczema treatments and the presence of abscesses make this less likely.

## 4. Conclusion

The diagnosis of HIES is inherently difficult due to the heterogeneous nature of its presentation. Many of the cutaneous and systemic features overlap with other immunodeficiency syndromes, chronic infections, or dermatologic conditions. In our case, the extensive pustular lesions, recurrent abscesses, and chronic respiratory symptoms initially led to misclassification as severe atopic dermatitis or recurrent bacterial infections. However, the combination of significantly elevated IgE levels, eosinophilia, and recurrent infections prompted consideration of a primary immunodeficiency, which ultimately led to a presumptive diagnosis of HIES. This case reinforces the importance of including immunological testing early in the diagnostic workup of patients with atypical or severe dermatologic and infectious symptoms.

Early recognition is essential to prevent complications, guide appropriate management, and offer genetic counseling. This case emphasizes the importance of maintaining a high index of suspicion in patients with recurrent infections, elevated IgE levels, and characteristic systemic features. Genetic analysis, particularly for STAT3 mutations, plays a vital role in confirming the diagnosis and differentiating between the various forms of HIES; it remains a challenge in a resource‐limited setup. Ongoing multidisciplinary care remains crucial to optimizing patient outcomes and quality of life.

## Author Contributions

Roshna Devi contributed to drafting the manuscript, data collection, and patient management. Huda Raja contributed to drafting the manuscript and data interpretation. Prithvi Raj contributed to the literature review and manuscript preparation. Muhammad Hassan Ahsan contributed to data analysis and manuscript revision. Nahid Raufi (corresponding author) contributed to conceptualization, supervision, and final review of the manuscript. Abdullah Nadeem contributed to writing original draft and editing.

## Funding

No funding was received for this research.

## Ethics Statement

The authors have nothing to report.

## Consent

Written informed consent was obtained from all the patients with identifiable information to publish their details in the manuscript.

## Conflicts of Interest

The authors declare no conflicts of interest.

## Data Availability

Data sharing is not applicable to this article as no datasets were generated or analyzed during the current study.

## References

[1] Hashemi H. , Mohebbi M. , Mehravaran S. , Mazloumi M. , Jahanbani-Ardakani H. , and Abtahi S. H. , Hyperimmunoglobulin E Syndrome: Genetics, Immunopathogenesis, Clinical Findings, and Treatment Modalities, Journal of Research in Medical Sciences. (2017) 22, no. 1, 10.4103/jrms.JRMS_1050_16, 53. 28567072 PMC5426098

[2] Mogensen T. H. , STAT3 and the Hyper-IgE Syndrome: Clinical Presentation, Genetic Origin, Pathogenesis, Novel Findings and Remaining Uncertainties, JAK-STAT. (2014) 2, no. 2, 10.4161/jkst.23435. PMC3710320 24058807

[3] Joshi A. Y. , Iyer V. N. , Hagan J. B. , St. Sauver J. L. , and Boyce T. G. , Incidence and Temporal Trends of Primary Immunodeficiency: A Population-Based Cohort Study, Mayo Clinic Proceedings. (2009) 84, no. 1, 16–22, 10.4065/84.1.16. 19121249 PMC2630110

[4] Chamlin S. L. , McCalmont T. H. , and Cunningham B. B. , et al.Cutaneous Manifestations of Hyper-IgE Syndrome in Infants and Children, Journal of Pediatrics. (2002) 141, no. 4, 572–575, 10.1067/mpd.2002.127503. 12378200

[5] Grimbacher B. , Holland S. M. , and Puck J. M. , Hyper-IgE Syndromes, Immunological Reviews. (2005) 203, no. 1, 244–250, 10.1111/j.0105-2896.2005.00228.x. 15661034

[6] DeWitt C. A. , Bishop A. B. , Buescher L. S. , and Stone S. P. , Hyperimmunoglobulin E Syndrome: Two Cases and a Review of the Literature, Journal of The American Academy of Dermatology. (2006) 54, no. 5, 855–865, 10.1016/j.jaad.2005.10.022. 16635666

[7] Woellner C. , Gertz E. M. , and Schäffer A. A. , et al.Mutations in STAT3 and Diagnostic Guidelines for Hyper-IgE Syndrome, Journal of Allergy and Clinical Immunology. (2010) 125, no. 2, 424–432, 10.1016/j.jaci.2009.10.059. 20159255 PMC2878129

[8] Ghareeb A. , Kakaje A. , Ghareeb A. , and Nahas M. A. , An Enormous Arteriovenous Malformation Presenting in a Child in Sacro-Gluteal Region and Managed Successfully by Recurrent Embolisation and Surgery, International Journal of Surgery Case Reports. (2020) 71, 244–249, 10.1016/j.ijscr.2020.05.010. 32492638 PMC7264987

[9] Kakaje A. and Awad R. , Behcet’s Disease Presenting With Primary Hypothyroidism, Adrenal Insufficiency, and Celiac Disease: A Case Report, Clinical Case Reports. (2020) 8, no. 12, 2350–2352, 10.1002/ccr3.3097. 33363738 PMC7752330

[10] Dave T. , Tashrifwala F. A. A. , Rangwala U. S. , and Hameed R. , Hyper-IgE Syndrome: A Case Report, Annals of Medicine & Surgery. (2024) 86, no. 2, 1205–1209, 10.1097/MS9.0000000000001670. 38333292 PMC10849427

